# Intercellular Communication-Related Molecular Subtypes and a Gene Signature Identified by the Single-Cell RNA Sequencing Combined with a Transcriptomic Analysis

**DOI:** 10.1155/2022/6837849

**Published:** 2022-05-16

**Authors:** Pin Guan, Wentao Cai, Ke Wu, Fan Jiang, Jinchan Wu, Xin Zhai, Min Zeng

**Affiliations:** ^1^Departments of Geriatrics Hainan General Hospital, Hainan Affiliated Hospital of Hainan Medical University, Haikou 570311, China; ^2^Spinal Surgery of Hainan General Hospital, Hainan Affiliated Hospital of Hainan Medical University, Haikou 570311, China; ^3^Department of Medical Oncology Hainan General Hospital, Hainan Affiliated Hospital of Hainan Medical University, Hakou 570311, China

## Abstract

**Background:**

The tumor microenvironment (TME) of lung adenocarcinoma (LUAD) comprise various cell types that communicate with each other through ligand-receptor interactions. This study focused on the identification of cell types in LUAD by single-cell RNA sequencing (scRNA-seq) data and screening of intercellular communication-related genes.

**Methods:**

The Gene Expression Omnibus (GEO) database (https://www.ncbi.nlm.nih.gov/geo) provided the RNA-seq data of LUAD patients in the GSE149655, GSE31210, and GSE72094 datasets. Quality control of the scRNA-seq data in GSE149655 was performed by the Seurat package (http://seurat.r-forge.r-project.org) for identifying highly variable genes for principal component analysis (PCA) and cell clustering. The CellPhoneDB (http://www.cellphonedb.org) was used for filtering intercellular communication-related ligand-receptor pairs. According to ligand and receptor expressions, LUAD samples were clustered using ConsensusClusterPlus (https://www.bioconductor.org/packages/release/bioc/html/ConsensusClusterPlus). Additionally, the identification of prognosis-related ligand and receptor genes was conducted along with the development of a risk prediction model by the least absolute shrinkage and selection operator (LASSO) Cox regression analysis.

**Results:**

This study identified twelve cell types in 8170 cells of LUAD tissues along with 219 ligand and receptor genes. LUAD was classified into three different molecular subtypes, among which cluster 3 (C3) had the longest overall survival (OS) time and cluster (C1) had the shortest OS time. In comparison with the other two molecular subtypes, it was observed that C1 had a higher rate of somatic mutations and lower levels of infiltrating immune cells and immune scores. Ten genes were screened from the total ligand and receptor genes to construct a risk model, which showed a strong prediction power in the prognosis of patients with LUAD.

**Conclusion:**

The results of this study revealed cell types specific to LUAD, which were classified into different molecular subtypes according to intercellular communication-related genes. A novel prognostic risk model was developed in this study, providing new insights into prognostic assessment models for LUAD.

## 1. Introduction

Lung cancer results in almost one-quarter of all cancer-related mortalities [1]. Lung adenocarcinoma (LUAD) is the most widely diagnosed histological subtype of lung cancer and usually progresses from human small airway epithelial cells and type II alveolar cells [2]. LUAD consists of adenocarcinoma in situ, atypical adenomatous hyperplasia, minimally invasive adenocarcinoma, and invasive adenocarcinoma [3]. The occurrence of LUAD is still increasing in people without smoking habit, and patients with topical and early-stage LUAD can take standard surgery. However, a large number of patients are typically diagnosed with advanced LUAD requiring conventional treatment such as chemotherapy combined with radiotherapy and face a high mortality risk, with a 5-year survival rate of about 15% [4]. Immunotherapies such as immune checkpoint inhibitors have proved to be highly effective in clinical trials. Nonetheless, many patients receiving immunotherapy will still develop immune resistance [5]. Intratumoral heterogeneity compromises the clinical efficacy of anticancer drugs, for instance, in the case of immunotherapy in cancer. It can be reasonably clarified that heterogeneity and different Spatio-temporal interactions among all cellular components within the tumor microenvironment (TME) results in cancer adaptation and treatment pressure [6]. Therefore, explaining the intratumoral heterogeneity of LUAD may help develop strategies to control or prevent drug resistance.

Recently, advances in single-cell sequencing technology help understand the heterogeneity of tumor cells. As an important factor in single-cell sequencing technology, single-cell RNA sequencing (scRNA-seq) technology is undoubtedly a helpful tool for understanding the properties of the variety of cell types within [7]. For instance, according to a recent report, scRNA-seq can examine cell clusters in tumor tissues, identify cell clusters causing drug resistance, and enable an analysis of the genetic features of drug-resistance cell clusters [8]. According to a previous report, the scRNA-seq has identified cell clusters with various functions and high invasive potential in heterogeneous primary glioblastoma [9]. scRNA-seq analysis in 15208 cells from a pair of primary and metastatic sites of collecting duct renal cell carcinoma identified cancer stem cell clusters that contribute to bone destruction in a positive feedback loop in bone metastasis microenvironment [10]. Other than characterizing the cellular composition of tumors, scRNA-seq can also improve the understanding of how the major cellular components cooperate to stimulate the emergent tumor behavior [11]. Currently, this exploratory area is also a significant part of cancer research. However, further research is required in this direction.

In this report, scRNA-seq data from 8170 cells of 2 LUAD samples and 2 normal samples were analyzed to explore the intratumor heterogeneity of LUAD by exploring cell clusters. Molecular subtyping of LUAD samples from The Cancer Genome Atlas (TCGA) (https://www.cancer.gov/about-nci/organization/ccg/research/structural-genomics/tcga), GSE31210, and GSE72094 was also performed by delineating gene expression profiles related to intercellular interactions among cell clusters. The risk assessment tool was constructed according to the intercellular communication-related genes for prognostic prediction of patients with LUAD. The work flow chart of this study is shown in Figure S1.

## 2. Materials and Methods

### 2.1. Single-Cell RNA Sequencing Data and RNA Sequencing Data of Lung Adenocarcinoma

The scRNA-seq data were provided by the Gene Expression Omnibus (GEO) database with the accession number GSE149655 [12] for the two primary LUAD samples and two normal samples.

We downloaded RNA-Seq data from 485 primary LUAD samples using the TCGA GDC API, and the GSE31210 [13] and GSE72094 [14] datasets were identified from the GEO database. After removing samples without records of survival status and survival times of less than 1 month, RNA-seq data of 226 and 386 LUAD samples in both datasets were collected, respectively.

### 2.2. Quality Control and Data Analysis of Single-Cell RNA Sequencing Data

Quality control and scRNA-seq data filtering were carried out to isolate cells with high-quality data utilizing the Seurat package (http://seurat.r-forge.r-project.org). Seurat objects were generated for each sample with the cell-by-gene count matrix using the CreateSeuratObject function (min.cells =3, min.features =250). We retained the cells of nFeature_RNA>600 and nFeature_RNA<6000 and mitochondrial gene percentage<35%. Raw counts were normalized based on the LogNormalize method in the NormalizeData function. Highly variable genes were recognized for principal component analysis (PCA). Additionally, important principal components (PCs) were identified using the jackStraw function. Subsequently, the top 50 PCs were used as input to the uniform manifold approximation and projection (UMAP). The resolution was set =0.2 and the cells were clustered by the Louvain algorithm with the FindClusters function. Finally, the FindAllMarkers function was used for the identification of the marker genes for a specific cluster.

### 2.3. Cell Type Identification

The CellMarker database (http://biocc.hrbmu.edu.cn/CellMarker/) [15] was used to download cell marker genes for lung tissue. Cellular clusters were annotated on the basis of well-known marker genes using the enricher function of the ClusterProfiler package [16] to identify the cell types to which various clusters belong.

### 2.4. Analysis of Intercellular Interactions

Intercellular communication was determined by the CellPhoneDB (version: 2.0) [17] applying the cluster annotation and counts from our scRNA-seq data to compute intercellular communication within the identified cell subtypes [18]. The default settings were used in the procedure. The higher ligand-receptor interaction scores reflected stronger potential interactions between cells.

### 2.5. Identification of Different Molecular Subtypes of LUAD Based on Ligand-Receptor Gene Expression

Spearman's correlation coefficients were calculated for the significant ligand-receptor pairs in the intercellular communication analysis on TCGA-LUAD dataset. This study screened the ligand-receptor pairs with Spearman's correlation coefficient greater than 0.3. The samples in the TCGA-LUAD dataset were clustered according to the gene expression of ligand-receptor pairs using ConsensusClusterPlus, where the parameters were set to metric distance =1 - Pearson correlation and bootstraps =500. The areas under the cumulative distribution function (CDF) curves at different k values were measured for determining the number of clusters.

### 2.6. Gene Set Enrichment Analysis and Functional Annotation

The ‘clusterProfiler' package was used to explore the potential molecular mechanisms of different molecular subtypes, including gene set enrichment analysis (GSEA), gene ontology (GO), and Kyoto Encyclopedia of Genes and Genomes (KEGG) pathways. The gene sets used in GSEA were all candidate gene sets from the Hallmark database. These outcomes were visualized by Dotplot as bubble plots.

### 2.7. Differences in the Tumor Microenvironment

To quantify the proportion of immune cells in the TME, differences in the proportion of immune cells in TME were assessed between different molecular subtypes in the three separate datasets, TCGA-LUAD, GSE31210, and GSE72094. Additionally, immune scores and stroma scores were also measured for the various subtypes in the three datasets using the ESTIMATE tool.

### 2.8. Construction and Validation of an Intercellular Communication-Related Risk Model Using Public Databases

To identify molecular subtype-related intercellular communication-related genes, differential expression analysis of ligand and receptor genes between molecular subtypes was performed to select ligand and receptor genes differentially expressed between molecular subtypes. Subsequently, the selected genes were subjected to survival-related univariate Cox regression analysis. A last absolute shrinkage was performed along with selection operator (LASSO) and multivariate Cox regression analyses to filter genes with the most prognostic value and to obtain a fit coefficient for every gene. Using the formula risk score = Ʃ (*β*i ∗ Expi), the risk scores of standardized patients in TCGA-LUAD, GSE31210, and GSE72094 datasets were measured and the optimal cut-off values obtained by the R package “survminer” were used to sort the patients into two groups (the high-risk group and the low-risk group). Kaplan-Meier analysis with a log-rank test was used for the assessment of patients' overall survival (OS) and the receiver operating characteristic (ROC) curve was time-dependent and plotted using the ‘timeROC' tool.

### 2.9. Statistical Analysis

The R software (version: 4.1.0) was used to carry out all statistical analyses. The RMaftools package presented the molecular mutations in different molecular subtypes via waterfall plots. Univariate and multivariate COX regression analyses helped assess the independent prognostic value of risk scores, and risk ratios (HR) and 95% confidence intervals (CI) are also granted for each variable. Unless otherwise stated, all parameters were default and p <0.05 represented a significant difference.

## 3. Results

### 3.1. Cellular Composition of LUAD Tumor Samples and Normal Samples

**Figure 1:**
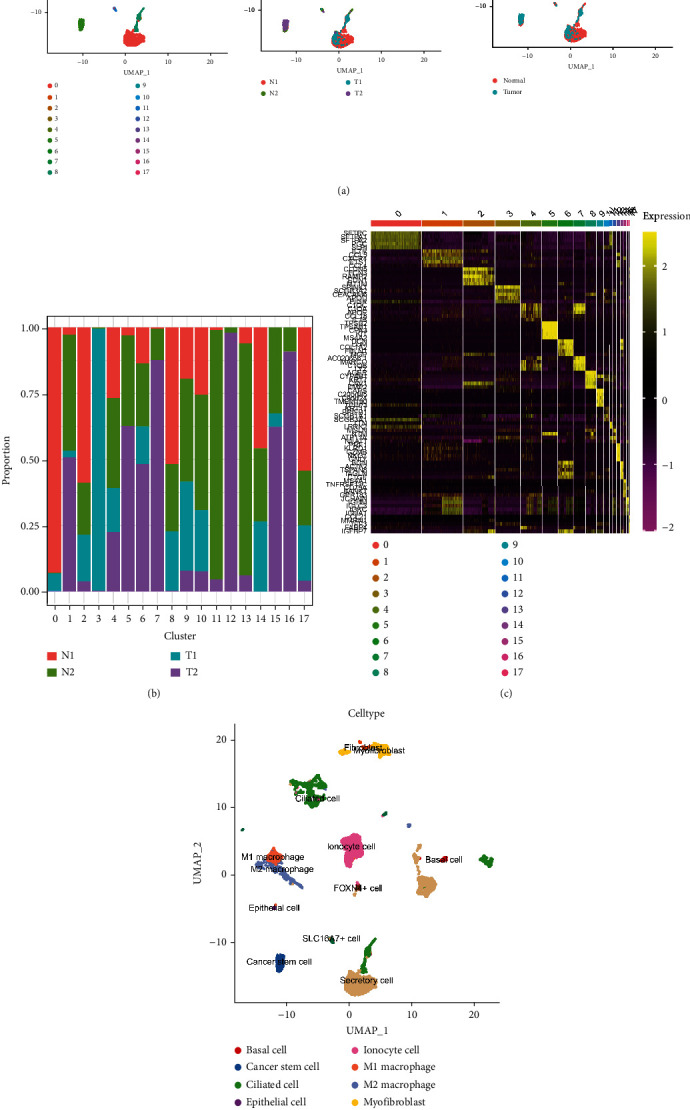
Cellular composition of LUAD tumor samples and normal samples. (a).UMAP plots of all cells used in this study are annotated according to cell cluster, sample source, and sample type. (b). Sample distribution of the 18 cell clusters. (c). Heat map of the most important differentially expressed genes in different cell clusters. (d). UMAP plots of scRNA-seq data for 12 cell types.

For understanding the intercellular heterogeneity of LUAD, the RNA-seq data were analyzed from a total of 12,254 cells taken from two LUAD samples and two normal samples. The relationship between the number of unique molecular identifiers (UMIs) and the number of mitochondrial genes or mRNAs were analyzed. The outcomes demonstrated that the number of UMIs did not correlate substantially with the percentage of mitochondrial genes, but was positively correlated with the number of mRNAs (Figure S2a and S2b). After initial quality control by Seurat, a total of 8170 cells were included in the analysis, including 4637 cells in normal tissues and 3533 cells in LUAD tumor tissues (Figure S2c and S2d). Among them, the genes highly variable were used for downstream analysis (Figure S2e). Unsupervised clustering analysis and the UMAP was performed for visualizing 18 cell clusters (Figure 1(a)). Notably, cluster 3, cluster 7, cluster 12, and cluster 16 were the predominant cell clusters present in normal tissues, whereas cluster 0, cluster 2, cluster 8, cluster 11, cluster 13, cluster 14, and cluster 17 were the predominant cell clusters present in LUAD tumor tissues (Figure 1(b)). Subsequently, cluster-specific marker genes were screened by differential gene expression analysis to define the identity of each cell cluster (Figure 1(c)). To demonstrate the cell identities represented by those clusters, 12 cell types were annotated according to the CellMarker package (Figure 1(d)), representing a wide variety of cell types including secretory cells, fibroblasts, epithelial cells, immune cells, and tumor cells (Table 1).

### 3.2. Global Comparative Analysis of Intercellular Communication in Lung Adenocarcinoma

**Figure 2:**
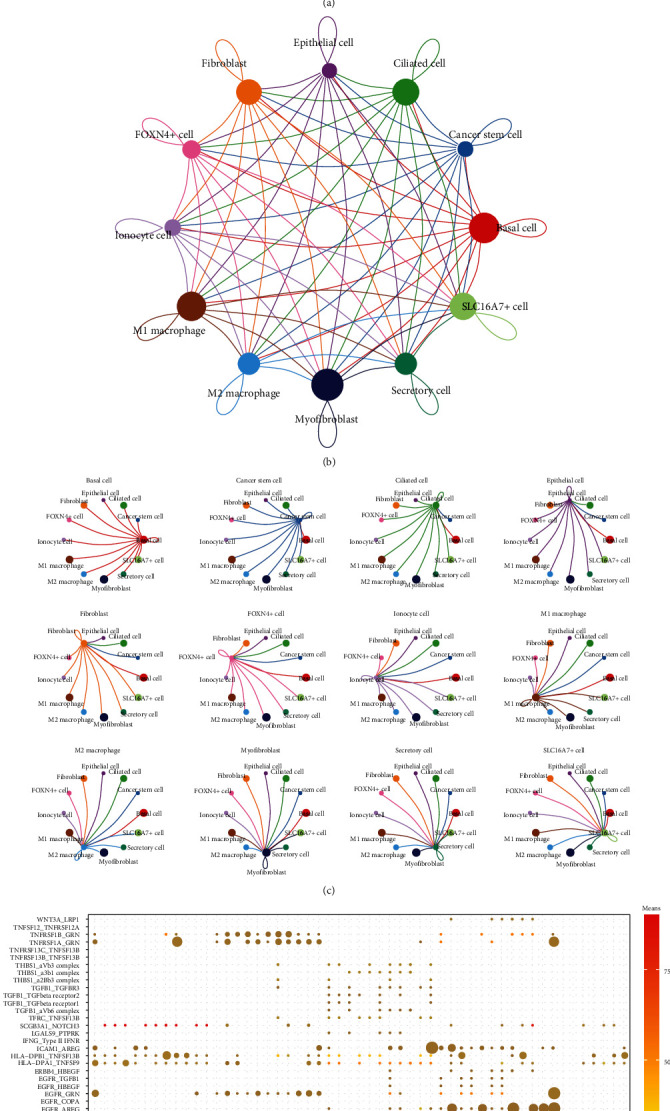
Network of 12 types of intercellular interactions in LUAD. A. Heat map showing the number of potential ligand-receptor pairs in 12 cell types. B and C. Interaction network of 12 cell types constructed by CellPhoneDB, where the thicker line indicates more interactions with other cell types. D. Important ligand-receptor pairs for Hedgehog, Notch, TGF*β*, WNT signaling, and EGFR signaling pathways during interactions between different cell types.

To better understand the interactions of various types of cells in the LUAD TME, the number of ligand-receptor pairs in 12 cell types in LUAD was analyzed with cellphoneDB. Among them, there were more ligand-receptor pairs between three cell types, including myofibroblast, basic cell, and myofibroblast (Figure 2(a)). The interaction network between the 12 cell types is illustrated in Figure 2(b). It was also observed that myofibroblast, basal cells, and M1 macrophages had the strongest intercellular interactions between clusters (Figure 2(c)). Additionally, genes in the Hedgehog, Notch, TGF*β*, WNT signaling, and EGFR signaling pathways, which are related to malignant progression of tumor, were selected for further investigating whether there were notable interactions between cell clusters. The bubble map indicated that the receptor TNFRSF1A and its corresponding ligand GRN played an important role in the communication between myofibroblasts and basal cells or cancer stem cells. Moreover, the ligand-receptor pair consisting of EGFR ligand and COPA or AREG was also the key ligand-receptor pair in the communication between myofibroblasts and cancer stem cells. Ligand-receptor pairs of EGFR and AREG also played a significant role in the communication of M1 macrophage with other types of cells (Figure 2(d)).

### 3.3. Three Molecular Subtypes of LUAD Based on Ligand and Receptor Genes

**Figure 3:**
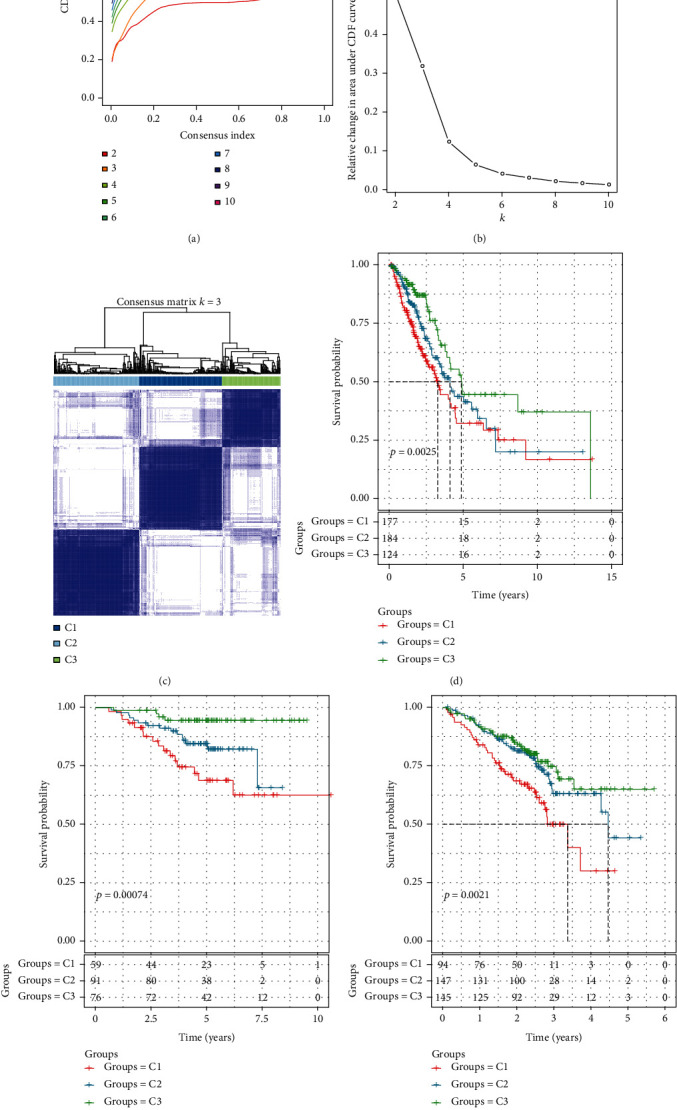
Classification of LUAD subtypes based on intercellular communication of ligand-receptor genes. A and B. CDF curves and delta area for the consensus cluster analysis of LUAD samples in TCGA; C. The consensus fraction matrix of TCGA samples when k =3; D. Survival analysis of three subtypes in TCGA; E. Kaplan-Meier curves for the three LUAD subtypes in GSE31210; F. Prognostic analysis of molecular subtypes of LUAD in GSE72094.

As the massive variations in the ligand-receptor pairs playing a leading role in interactions between different cell types, ligand-receptor pair genes that are involved in major interactions in different cell types were extracted and used to classify the molecular subtypes of the 485 LUAD samples in TCGA. The 3-cluster solution corresponded to the largest cluster number that induced the least incremental change in the area under the CDF curves while keeping the maximal consensus within clusters and the minimal rate of ambiguity in cluster assignments (Figures 3 (a) and 3(b)). In particular, LUAD was classified into three molecular subtypes namely, cluster 1 (C1), cluster 2 (C2), and cluster 3 (C3) (Figure 3(c)). In terms of OS time, C3 had the longest OS time, while C1 had the shortest OS time both in the TCGA and in the GSE31210 and GSE72094 datasets. There were major differences in OS among the three subtypes (Figures 3(d)–3(f)).

### 3.4. Molecular Characteristics of the Three Molecular Subtypes

**Figure 4:**
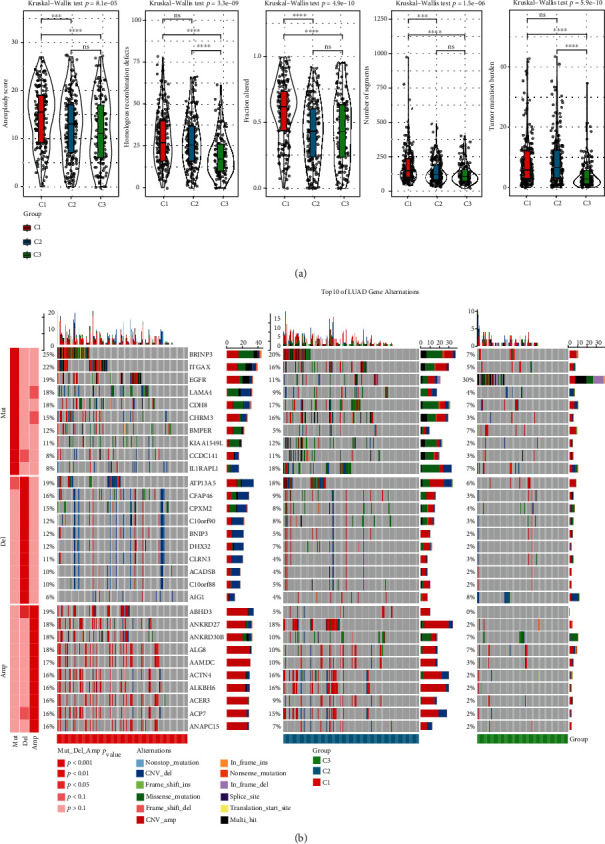
Analysis of genetic alterations in the three subtypes. (a). Aneuploidy scores, homologous recombination defects, fraction altered, number of segments, and tumor mutation burden for the three molecular subtypes (Kruskal-Wallis test). (b). The top panel represents mutation rates in the three subtype samples; the middle panel represents copy number deletions; the bottom panel represents copy number amplifications (Fish test). ∗ P <0.05, ∗∗ P <0.01, ∗∗∗ P <0.001, ∗∗∗∗ P <0.0001.

For observing the molecular features of different subtypes, we performed the genomic damage analysis on the three molecular subtypes. The three subtypes showed greatly variable aneuploidy scores, homologous recombination defects, fraction altered, number of segments, and tumor mutation burden, with the C1 subtype indicating higher levels of these five indicators (Figure 4(a)). Among the mutations in the three subtypes of TCGA, substantially higher mutation rates were observed for BRINP3, ITGAX, and LAMA4 in C1 than in C2 and C3. The mutation rate of the classical cancer-related gene EGFR was substantially higher in C3 than in C1 and C2. Additionally, the mutation rate of the gene IL1RAPL1 was much higher in C2 than in C1 and C3 and C1 demonstrated the most prevalent copy number amplification and deletion in all three subtypes (Figure 4(b)).

### 3.5. Functional Analysis of Three Molecular Subtypes

**Figure 5:**
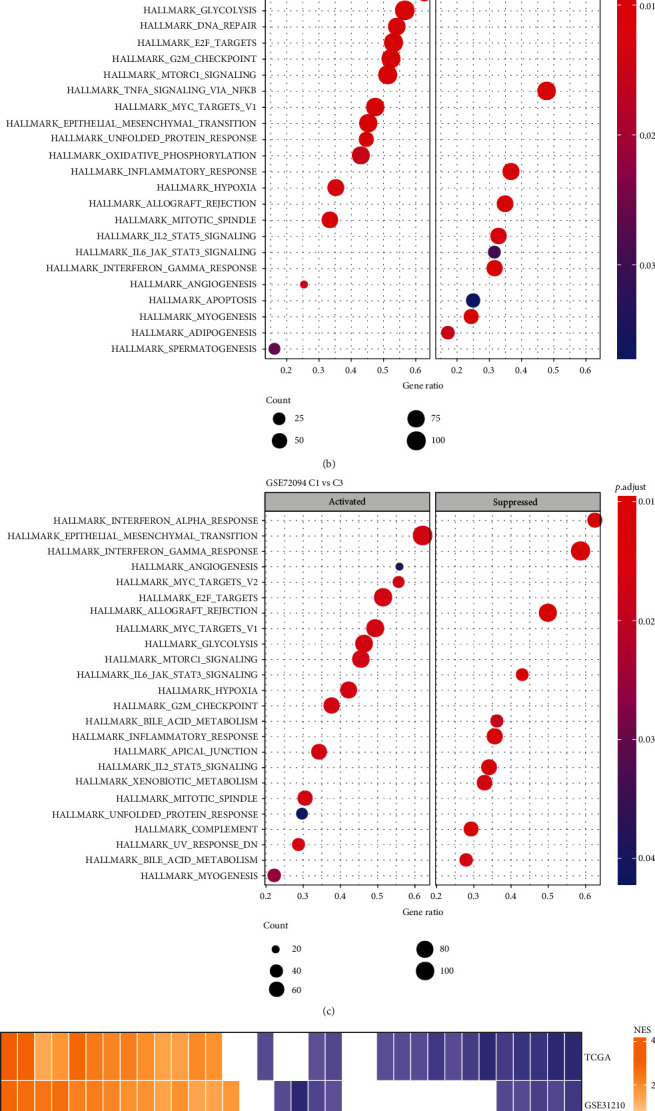
GSEA analysis of molecular subtypes. A-C. GSEA analysis of C1 vs. C3 in TCGA, GSE31210, and GSE72094 datasets. D. The heat map demonstrating normalized enrichment scores (NESs) of Hallmark pathways calculated by comparing C1 with C3. E. Radar plots indicating NESs of Hallmark pathways calculated through a GSEA of C 1 vs. C3 and of C2 vs. C3 in TCGA, GSE31210, and GSE72094 datasets.

Afterward, the potential biological pathways involved in each molecular subtype in the TCGA, GSE31210, and GSE72094 datasets were analyzed. In TCGA, 13 activated pathways were detected along with 15 inhibited pathways in the C1 subtype in comparison with the C3 subtype, and the activated pathways in C1 included oncogenic pathways such as MYC targets, E2F targets, and G2M checkpoint. The inhibited P53 pathway and apoptosis were well recognized pathways (Figure 5(a)). Furthermore, the MYC targets, E2F targets, and G2M checkpoint were also greatly activated in the C1 subtype of the GSE31210 and GSE72094 datasets (Figure 5(b) and 5(c)). In addition, consistent activation and inhibition of pathways between subtypes for the different LUAD datasets were demonstrated (Figure 5(d)). Overall, the C1 subtype showed an activated state in the cell cycle and an inhibited state in the immune regulatory pathway (Figure 5(e)). Therefore, it was hypothesized that these ligand and receptor genes used to classify molecules may play major regulatory roles in the immunosuppressive microenvironment and the cell cycle.

### 3.6. Tumor Microenvironment Characteristics of Different Molecular Subtypes

**Figure 6:**
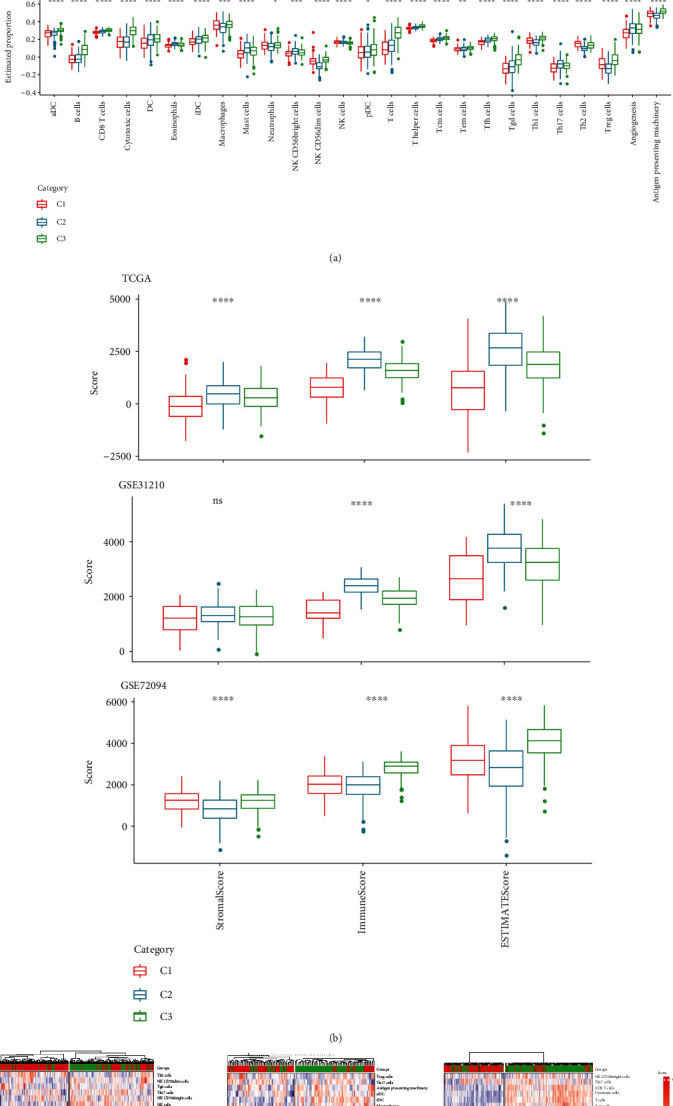
Assessment of TME characteristics of three molecular subtypes in different datasets. (a). TME cell content of the three molecular subtypes in the TCGA, GSE31210, and GSE72094 datasets evaluated as per the marker genes in immune cells. (b). Stromal score, immune score, and ESTIMATE score of the three molecular subtypes calculated using the ESTIMATE algorithm in the TCGA, GSE31210, and GSE72094 datasets. (c). Unsupervised hierarchical clustering of immune cell scores based on C1 and C3.

This study found the cell infiltration within TME in LUAD molecular subtypes grouped based on intercellular communication-related genes. The cell levels in TME of the three subtypes in TCGA, GSE31210, and GSE72094 datasets were first observed based on marker genes [19] in immune cells and 26 TME cells were found to be much different among the three molecular subtypes, such as dendritic cells (DC), B cells, T cells, cytotoxic cells, eosinophils, macrophages, mast cells, and natural killer cells (NK cells). Furthermore, the three molecular subtypes differed greatly in terms of angiogenesis and antigen-presenting machinery, with C3 scoring much higher on these two measures than C1 (Figure 6(a)). In addition, the stromal score, immune score, and ESTIMATE score generated by the ESTIMATE algorithm indicated that C3 had a substantially higher immune score and ESTIMATE score compared to C1 in all the three datasets (Figure 6(b)). Therefore, C3 showed relatively high immune cell infiltration. By performing an unsupervised hierarchical clustering of immune cell scores for C1 and C3, patients were classified into two groups, namely, the high- and low-immune infiltration groups. It was observed that the majority of the C3 samples were in the high-immune infiltration group, whereas most C1 belonged to the low-immune infiltration group (Figure 6(c)).

### 3.7. Identification of Ligand and Receptor Genes Associated with the Prognostic Prediction of LUAD Patients

**Figure 7:**
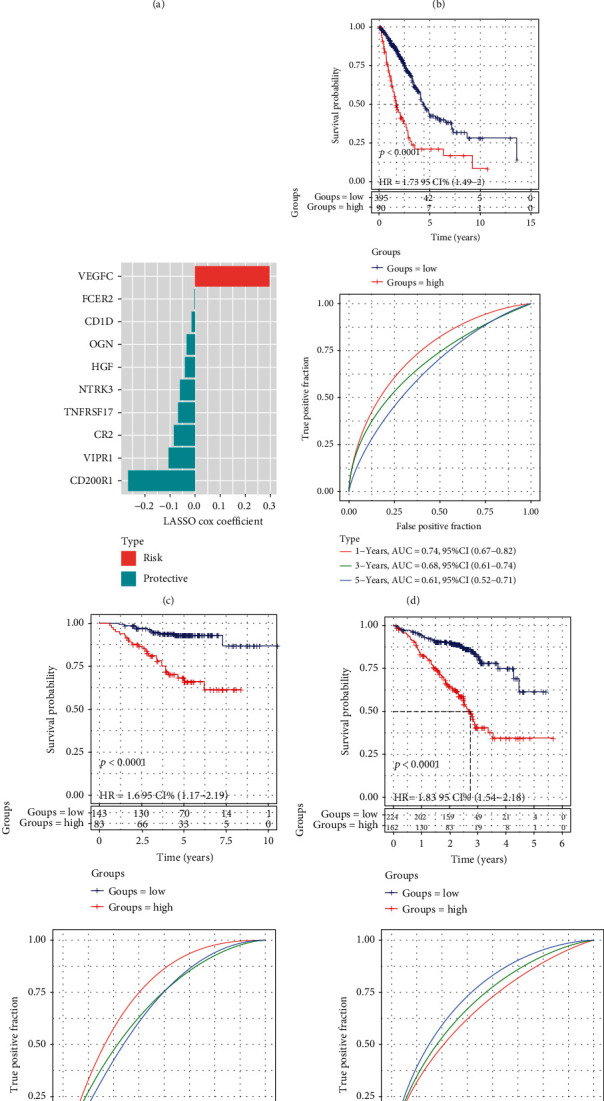
Prognostic risk model of the ten genes associated with intercellular communication in the training dataset and external validation datasets.A and B. LASSO Cox regression analysis according to 32 ligand and receptor genes. C. LASSO coefficient distributions for 10 hub ligand and receptor genes. D-F. Kaplan-Meier curves and corresponding areas under ROC curve for LUAD samples in TCGA, GSE31210, and GSE72094 datasets.

Finally, differential expression of ligand and receptor genes was analyzed in the three subtypes, and it was observed that 180 of the ligand and receptor genes were differentially expressed in the three molecular subtypes. Univariate Cox regression analysis identified 32 genes among the 180 ligands and receptors correlated with the survival of LUAD patients. LASSO Cox regression analysis based on the 32 genes helped identified10 hub ligand and receptor genes, and regression coefficients were measured for individual genes (Figures 7(a) and 7(b)). VEGFC was the risk factor of LUAD, while the FCER2, CD1D, OGN, HGF, NTRK3, TNFRSF17, CR2, VIPR1, and CD200R1 were the protective factors of LUAD (Figure 7(c)). After calculating the risk score for each sample in the training set, a survival analysis was carried out and the results indicated that high-risk LUAD patients had worse OS as compared to the low-risk LUAD patients. Among them, high-risk samples mainly came from C1 subgroup, a small amount from C2 subgroup, and low-risk samples mainly came from C3 and most of C2 subgroup (Figure S3a). ROC curves were plotted and the one-, three-, and five-year areas under the curve (AUC) were noted to be 0.74, 0.68, and 0.61, respectively (Figure 7(d)). Kaplan-Meier curves were plotted in the two GEO validation datasets, and the outcomes highlighted that high-risk scores were greatly linked to shorter OS for LUAD patients in the GSE31210 and GSE72094 validation datasets. The AUC values for the ROC used to assess the risk model were higher in both independent validation datasets (Figures 7(e) and 7(f)).

## 4. Discussion

The TME consists of various cell types, and the interactions between these different cell types are correlated with tumorigenesis, tumor progression, treatment resistance, and the stroma of immune infiltration [20]. These various cell types interact with each other via ligand-receptor interactions. Considering the significance of such interactions in patients' treatment outcomes, therapies targeted towards intercellular interactions have become increasingly popular in clinical practice [11]. However, to date, enough research has not been done to identify genes linked to intercellular communication.

Numerous tools have been established using scRNA-seq data for the analysis of intercellular ligand-receptor interactions, and therefore reveal intercellular communication in TME [17, 21, 22]. In this report, the scRNA-seq data was provided by GSE149655 for two LUAD samples and two normal samples. Analysis of scRNA-seq data from the obtained 8,170 cells revealed that 18 cell clusters belonged to 12 cell types, involving broad cell types such as secretory cells, fibroblasts, epithelial cells, immune cells, and tumor cells. Interaction networks were created for the 12 cell types using cellphoneDB, a tool the most widely utilized for studying intercellular interactions. Moreover, in this study, ligand-receptor pairs that greatly interact in the 12 cell types of communication were extracted and we further divided LUAD into three molecular subtypes according to these intercellular communication-related molecules.

Among the three molecular subtypes, C3 showed the longest OS time, followed by C2 and C1. Single nucleotide variants have been reported to increase progressively throughout progressive lesions from lung tumor precursors to LUAD [23]. Furthermore, pre-malignant lesions in invasive LUAD show immune activation and anti-tumor immune deficits in more advanced lesions [24]. It was observed in this study that C1 displayed more severe DNA damage and somatic cell alterations in the three molecular subtypes. Moreover, the C1 subtype showed an activated state in the cell cycle, while the immune regulation was suppressed. The MYC targets, E2F targets, and G2M checkpoint, which are all hub pathways regulating cell proliferation [25], were activated in the subtype C1. Therefore, these results indicated that the C1 subtype was more malignant, leading to the shortest OS time.

Finally, the intercellular communication-related scoring model was constructed by screening ligand and receptor genes that were all differentially expressed in the three subtypes. Furthermore, the effects of some of the genes in the model on tumors have been highlighted in several reports. The VEGFC, an activator of lymphangiogenesis, has been observed to promote all aspects of oncogenicity by autocrine regulation [26] and is correlated with poor prognosis in patients with LUAD [27]. The CD1D molecules may stimulate anti-tumor immune responses by presenting tumor-derived lipid and glycolipid antigens to T cells and NKT cells [28]. OGN plays an oncogenic role in the progression of both breast cancer [29] and colorectal cancer [30]. The Overexpression of HGF is linked to a poor prognosis of patients in many solid tumors, such as lung cancer, head and neck, gastrointestinal, breast, and cervical cancers [31]. Similarly, NTRK3 is a tumor suppressor gene [32]. The VIPR1 gene is downregulated at the level of LUAD cells and plays a key tumor suppressor role in the progression of LUAD [33]. The mRNA expression of CD2000R1 was a favorable prognostic factor in patients with non-small cell lung cancer [34]. The analysis in this study suggested that the VEGFC was a risk factor in LUAD, whereas the rest of the nine genes were protective factors, which aligned with the results shown in the mentioned previously reported research. In addition, we also compared the expression relationship of these 10 genes. It can be observed that most of these genes showed significant positive correlation. It is worth mentioning that VEGFC had the weakest correlation with other genes, suggesting that VEGFC may be independently involved in the occurrence and development of tumors (Figure S3b). Comparison on the relationship between these genes and immune cell infiltration in the immune microenvironment demonstrated that these 10 genes were highly correlated with immune cell infiltration in 22. Among them, CD2000R1，CD1D,FCER2 and TNFRSF17 were mainly positively correlated with immune infiltrating cells, VIPR1 and VEGFC were mainly negatively correlated with immune infiltrating cells (Figure S3c). These results showed that these 10 genes were highly involved in the regulation of immune microenvironment, and they may play different roles in different temporal and spatial states. In conclusion, according to the analysis of scRNA-seq data from LUAD samples, this study characterized heterogeneous cell clusters in LUAD, providing a deeper understanding of the potential intercellular interactions in the TME of LUAD. Moreover, ligand-receptor genes that determined intercellular interactions were identified, different molecular subtypes of LUAD were clarified, and a novel intercellular communication-related risk model was developed. Our results not only improved the current classification of LUAD at the cellular level, but also provided a potential predictive model for the prognosis of LUAD patients.

## Figures and Tables

**Table 1:** Cell types represented by 18 cell clusters.

Cell type	Cluster
Secretory cell	0
Ionocyte cell	1
Ciliated cell	2
Secretory cell	3
M2 macrophage	4
Cancer stem cell	5
Myofibroblast	6
M1 macrophage	7
Ciliated cell	8
Ciliated cell	9
Secretory cell	10
SLC16A7+ cell	11
Basal cell	12
FOXN4+ cell	13
Fibroblast	14
Epithelial cell	15
SLC16A7+ cell	16
SLC16A7+ cell	17

## Data Availability

All data generated or analysed during this study are included in this article.

## Supplementary Materials

Supplementary 1 Figure S1: Work flow chart.

Supplementary 2 Fig. S2 Quality control of single cells in LUAD tissues and in normal tissues. A. Relationship between the percentage of mitochondrial genes and mRNA reads. B. Relationship between the number of mRNA and mRNA reads. C. Scatter plot before quality control illustrating the number of genes, UMI, and percentage of mitochondrial genes in each cell type from four samples. D. Scatter plot after quality filtering showing the number of genes, UMI, and percentage of mitochondrial genes in each cell type from four samples. E. Scatterplot of the top 2000 highly variable genes.

Supplementary 3 Fig. S3 Relationship between gene expression and immunity. A: The intersection relationship between the three subtypes and high and low risk samples. B: Heat map of expression correlation among 10 genes. C: Heat map of the correlation between the expression of 10 genes and the scores of 22 immune infiltrating cells.∗Indicates P <0.05, ∗∗ indicates P <0.01, ∗∗∗ indicates P <0.001.
