# Supplementary figures and images for: Intercellular Communication-Related Molecular Subtypes and a Gene Signature Identified by the Single-Cell RNA Sequencing Combined with a Transcriptomic Analysis
Source: Dis Markers. 2022 May 16;2022:6837849. doi: 10.1155/2022/6837849 (PMC9127593; doi:10.1155/2022/6837849)

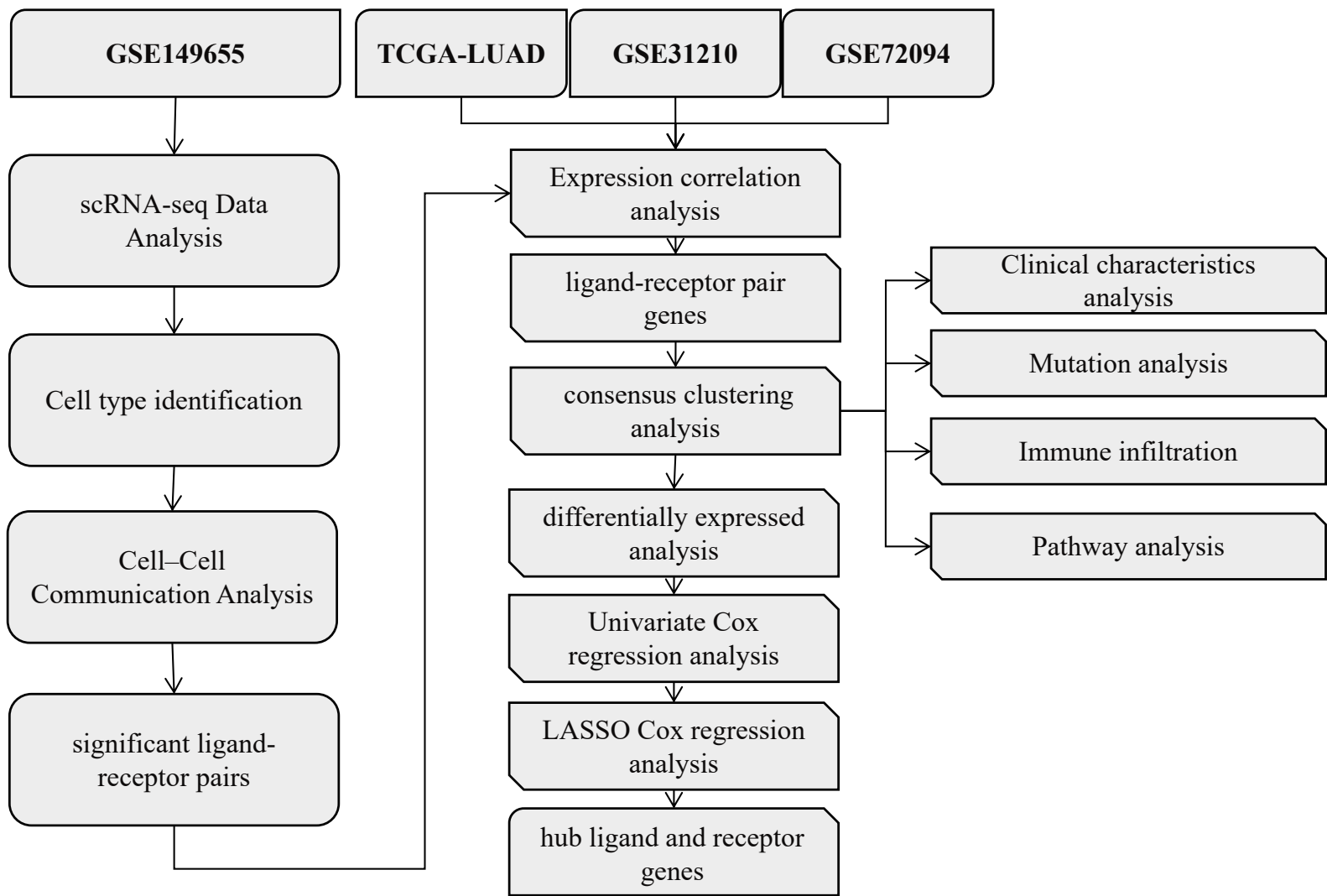

Supplement: Supplementary 1 — Figure S1: Work flow chart. [file 6837849.f1.pdf]

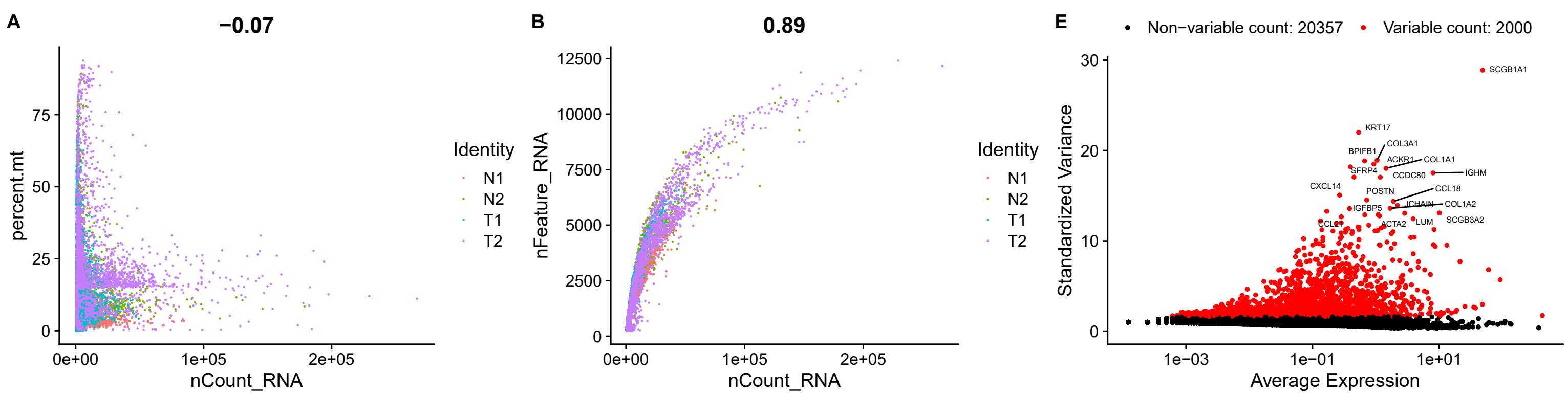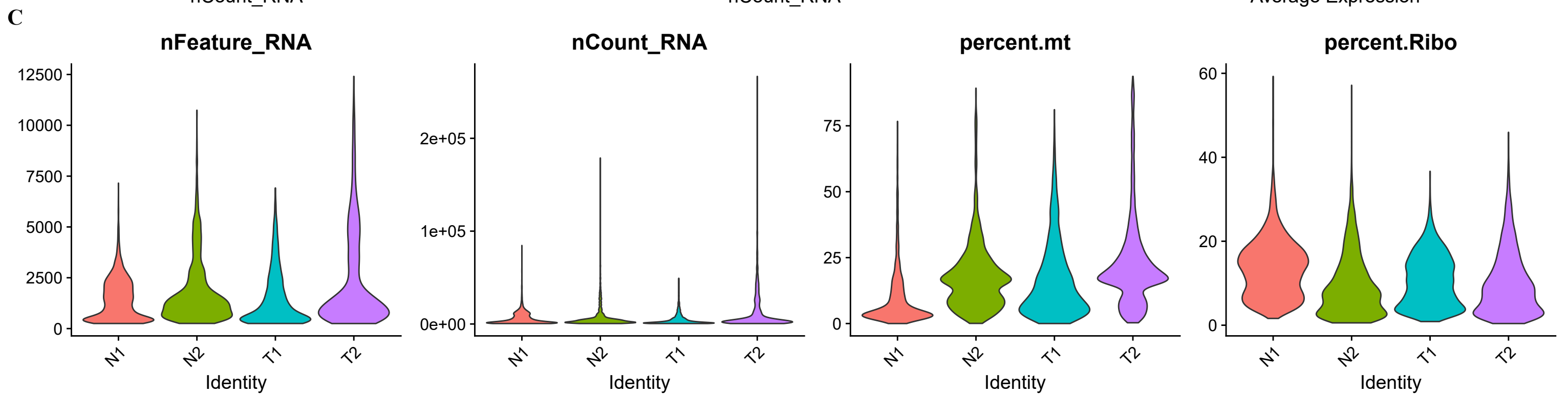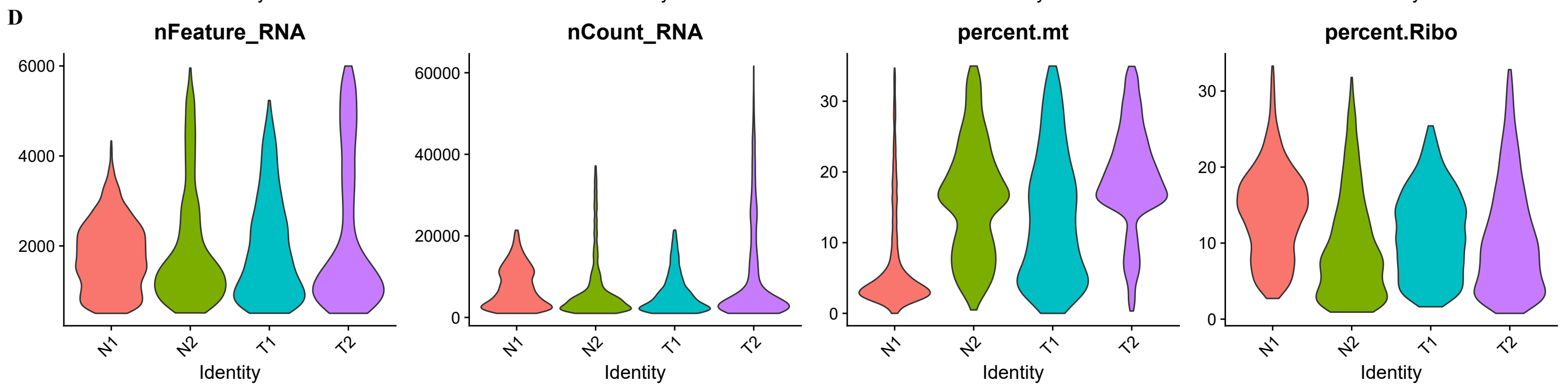

Supplement: Supplementary 2 — Fig. S2 Quality control of single cells in LUAD tissues and in normal tissues. A. Relationship between the percentage of mitochondrial genes and mRNA reads. B. Relationship between the number of mRNA and mRNA reads. C. Scatter plot before quality control illustrating the number of genes, UMI, and percentage of mitochondrial genes in each cell type from four samples. D. Scatter plot after quality filtering showing the number of genes, UMI, and percentage of mitochondrial genes in each cell type from four samples. E. Scatterplot of the top 2000 highly variable genes. [file 6837849.f2.pdf]

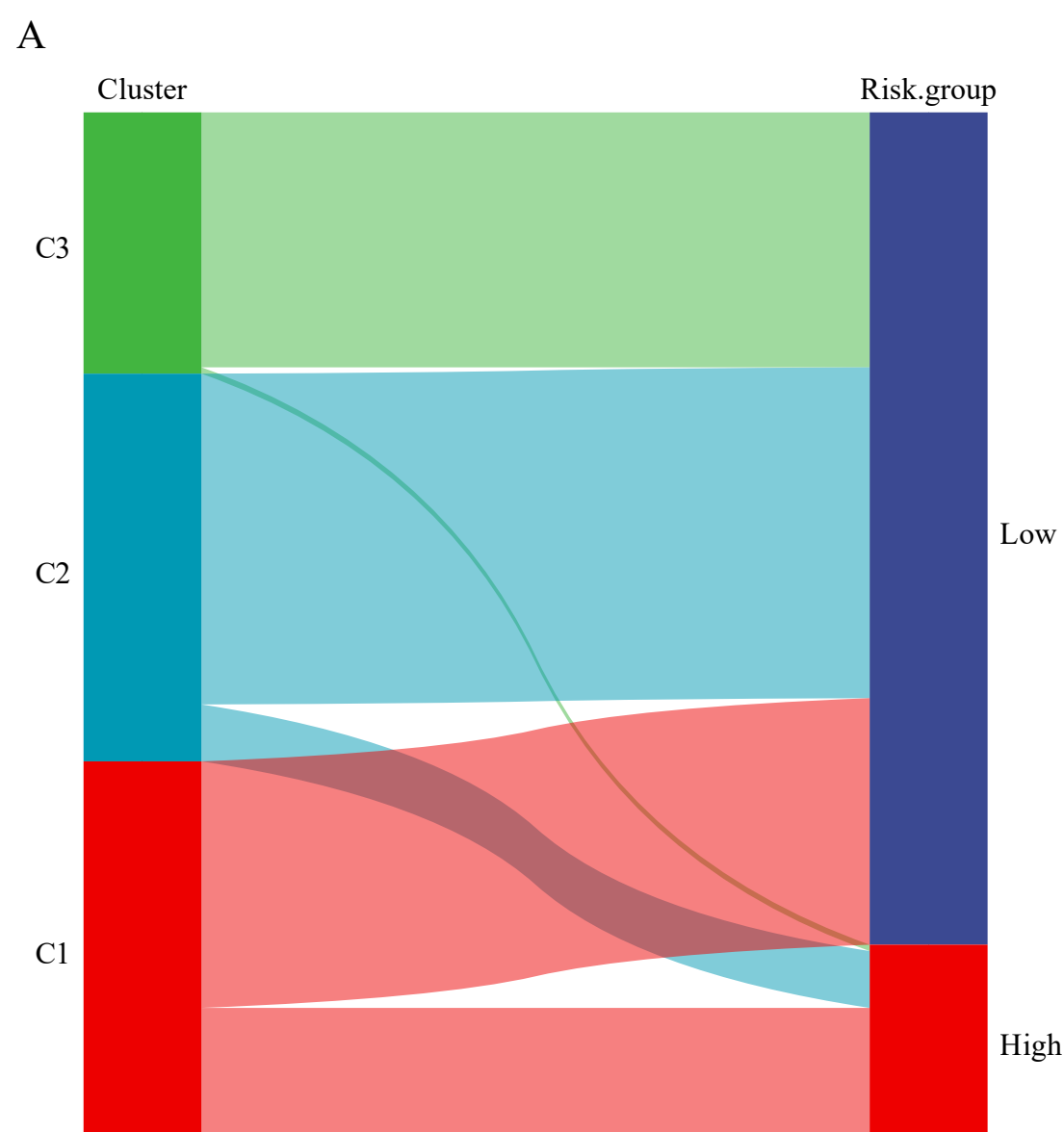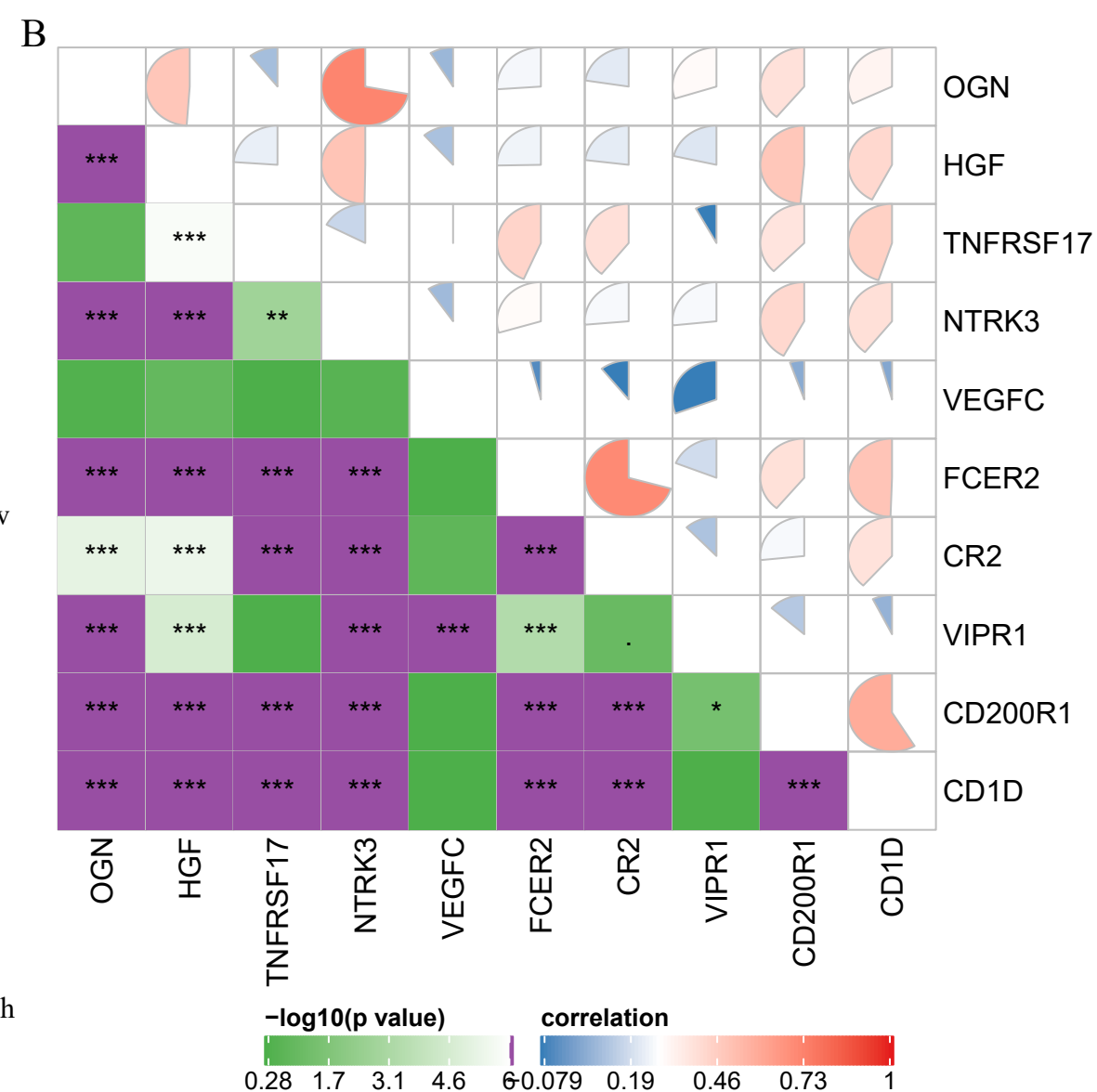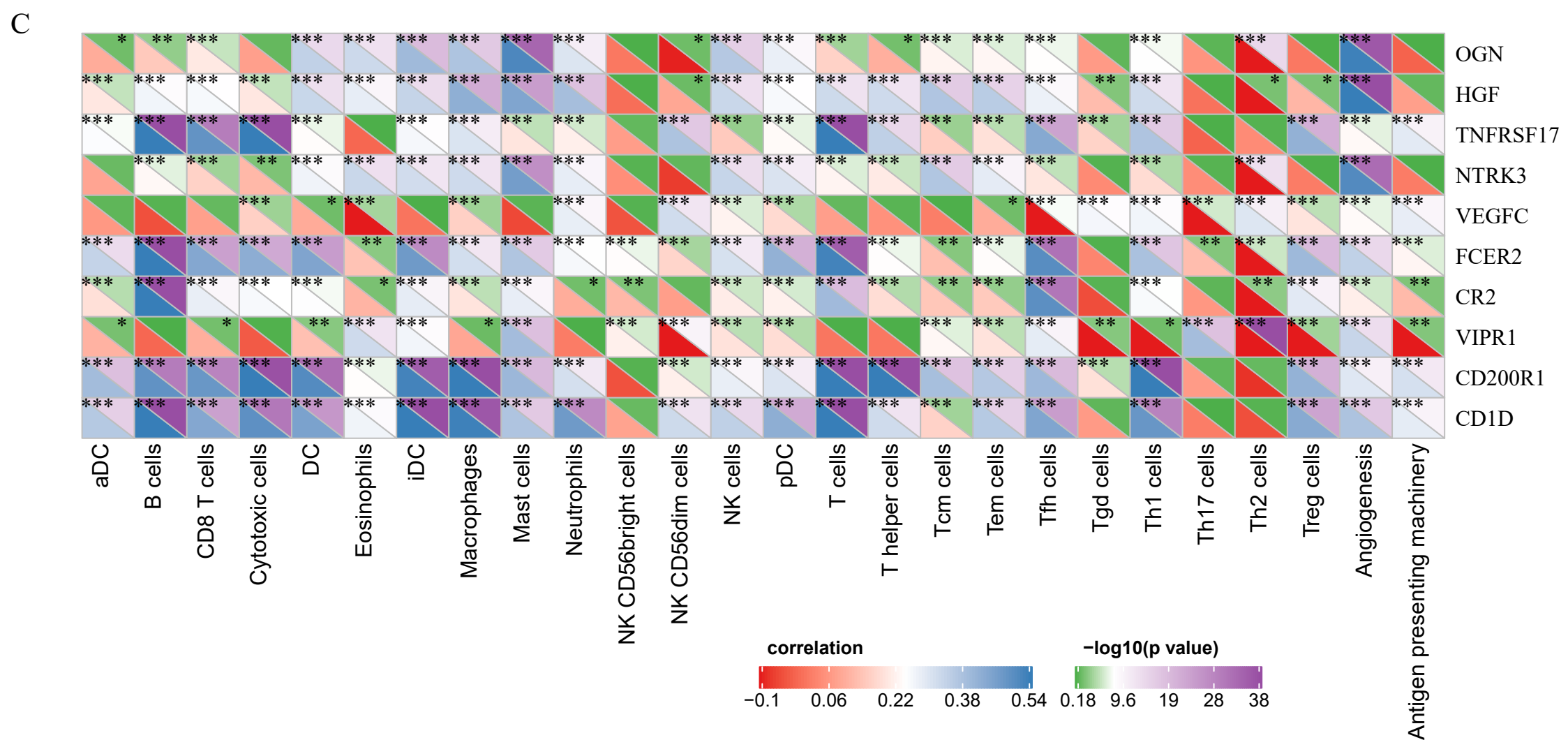

Supplement: Supplementary 3 — Fig. S3 Relationship between gene expression and immunity. A: The intersection relationship between the three subtypes and high and low risk samples. B: Heat map of expression correlation among 10 genes. C: Heat map of the correlation between the expression of 10 genes and the scores of 22 immune infiltrating cells.∗Indicates P <0.05, ∗∗ indicates P <0.01, ∗∗∗ indicates P <0.001. [file 6837849.f3.pdf]
